# Home medicines reviews following acute coronary syndrome: study protocol for a randomized controlled trial

**DOI:** 10.1186/1745-6215-13-30

**Published:** 2012-04-02

**Authors:** Daniel DL Bernal, Leanne Stafford, Luke RE Bereznicki, Ronald L Castelino, Patricia M Davidson, Gregory M Peterson

**Affiliations:** 1 Unit for Medication Outcomes Research and Education (UMORE), School of Pharmacy, University of Tasmania (UTAS), Sandy Bay Campus, Tasmania 7001, Australia; 2 Centre for Cardiovascular and Chronic Care, Faculty of Nursing, Midwifery and Health, University of Technology Sydney (UTS), Sydney 2007, Australia

**Keywords:** Acute coronary syndrome, Home medicines review, Medication adherence

## Abstract

**Background:**

Despite continual improvements in the management of acute coronary syndromes, adherence to guideline-based medications remains suboptimal. We aim to improve adherence with guideline-based therapy following acute coronary syndrome using an existing service that is provided by specifically trained pharmacists, called a Home Medicines Review. We have made two minor adjustments to target the focus of the existing service including an acute coronary syndrome specific referral letter and a training package for the pharmacists providing the service.

**Methods/Design:**

We will be conducting a randomized controlled trial to compare the directed home medicines review service to usual care following acute coronary syndromes. All patients aged 18 to 80 years and with a working diagnosis of acute coronary syndrome, who are admitted to two public, acute care hospitals, will be screened for enrolment into the trial. Exclusion criteria will include: not being discharged home, documented cognitive decline, non-Medicare eligibility, and presence of a terminal malignancy. Randomization concealment and sequence generation will occur through a centrally-monitored computer program. Patients randomized to the control group will receive usual post-discharge care. Patients randomized to receive the intervention will be offered usual post-discharge care and a directed home medicines review at two months post-discharge. The study endpoints will be six and twelve months post-discharge. The primary outcome will be the proportion of patients who are adherent to a complete, guideline-based medication regimen. Secondary outcomes will include hospital readmission rates, length of hospital stays, changes in quality of life, smoking cessation rates, cardiac rehabilitation completion rates, and mortality.

**Discussion:**

As the trial is closely based on an existing service, any improvements observed should be highly translatable into regular practice. Possible limitations to the success of the trial intervention include general practitioner approval of the intervention, general practitioner acceptance of pharmacists' recommendations, and pharmacists' ability to make appropriate recommendations. A detailed monitoring process will detect any barriers to the success of the trial. Given that poor medication persistence following acute coronary syndrome is a worldwide problem, the findings of our study may have international implications for the care of this patient group.

**Trial registration:**

Australian New Zealand Clinical Trials Registry ACTRN12611000452998

## Background

Optimal medication management is critical to improving outcomes following acute coronary syndrome (ACS), yet prescribing practices and patient behaviors often fall below recognized targets [1-3]. Research demonstrates that the prescribing of guideline-based medications following ACS, including antithrombotics, beta-blockers, angiotensin-converting enzyme inhibitors, and statins, has improved over recent years [4] but is not yet ideal [3]. When patients with ACS leave hospital and return to the community, however, a more significant problem becomes apparent due to the premature discontinuation of guideline-based therapies by patients and/or clinicians [1-3]. Furthermore, the majority of the morbidity and mortality that occurs in the early post-discharge period can be attributed to a failure to appropriately use these existing medications [5-8]. For example, premature medication discontinuation, when measured at one month post-discharge in a myocardial infarction population, decreased 12-month survival by 8% [2]. While, understandably, much of the focus on improving patient outcomes following ACS has centered on the acute phase of care, it is crucial that more attention is paid to the development of new, post-discharge strategies to improve outcomes following hospital discharge.

Similar to other developed countries [2], poor medication persistence following ACS has been recognized as an unresolved problem in Australia [3,9]. A recent Australia-wide quality improvement study, called Discharge Management of Acute Coronary Syndrome (DMACS) utilized an education-based intervention to improve in-hospital, guideline-based prescribing and this resulted in a significant improvement in the proportion of patients prescribed four guideline-based medications at discharge, from 57% at baseline to 69% post-intervention (*P *< 0.0001) [3]. A telephone follow-up of the post-intervention group, however, revealed a 17% decline in the proportion of patients taking all four guideline-based medications at three months post-discharge. This equated to a 66% loss of the improvement in prescribing that was recorded at discharge, by just three months post-discharge and suggested that the intervention was effective at improving in-hospital prescribing, but there was little effect on long-term medication use. The authors noted in their discussion that further community follow-up once the patients were settled in their homes could improve the poor rates of post-discharge medication persistence.

### ACS post-discharge services

Cardiac rehabilitation has been continually shown to improve multiple outcomes following ACS, including mortality [10]. As such, the DMACS study also aimed to improve referral rates and uptake of cardiac rehabilitation. While post-intervention referral rates significantly increased from 67% to 73% (*P *= 0.001), attendance rates remained unchanged at just 33% [3], which is in line with the low worldwide attendance rates for cardiac rehabilitation programs [11]. A Cochrane review has highlighted the potential for structured interventions to successfully increase cardiac rehabilitation uptake; however, interventions aimed at improving cardiac rehabilitation completion rates have generally proven less successful [12]. Although the benefits of cardiac rehabilitation are well-established, attendance and completion rates remain low, and evidence-based strategies to overcome these problems and increase access to the service are yet to be recognized.

Cardiac rehabilitation programs are designed to increase adherence to both pharmacological and non-pharmacological therapies [13]. However, there have been few evaluations of the specific effect of these programs on medication adherence. One study of a relatively high-intensity cardiac rehabilitation program was able to demonstrate an improvement in medication adherence at three months post-discharge [14], but despite these initial improvements, the benefit was short-lived, with both treatment and control groups reporting the same level of medication adherence at six months post-discharge. There is a clear need for a more intensive multidisciplinary approach to the post-discharge management of ACS, including interventions specifically directed at improving medication management. These interventions should be designed to both complement and promote the existing cardiac rehabilitation programs, as well as being accessible to the large majority of patients who currently find reasons not to attend cardiac rehabilitation.

The Home Medicines Review (HMR) program is an existing community-based service that has the potential to be tailored to meet the needs of patients recently discharged from hospital following ACS. Although this potential has not been fully realized in the past [15], HMRs may be used to particularly target patients' persistence and adherence to guideline-based medications following ACS. The HMR service currently involves general practitioner (GP) referral of patients to an HMR-accredited pharmacist, often through a community pharmacist liaison [16]. The accredited pharmacist will visit the patient in their home, discuss their medication taking habits, and provide education or adherence interventions where required. Following the home visit the accredited pharmacist writes a report for the GP noting their observations and any clinical adjustments that could be made to the patient's medication regimen. Based on this report, the GP is expected to complete an agreed management plan, selecting the accredited pharmacist's recommendations which they agree to implement and/or follow up. The service is currently available in Australia, free of charge to Australian citizens under funding arrangements through the public health system, Medicare Australia. The GP, accredited pharmacist, and community pharmacist involved with an HMR service are paid by way of reimbursement from Medicare Australia. One of the eligibility criteria for HMRs is recent hospital discharge [16] and the HMR service has been demonstrated to be feasible as a post-discharge service for patients following ACS in the Australian healthcare setting [17]. However, the effect of this service on clinical outcomes following ACS is unknown.

### Justification for this trial

Programs allowing pharmacists to provide formal medication review services exist across many countries. Although there are some regional differences in the structure of these programs, they are generally designed to improve the quality use of medicines and minimize the potential for medication-related harm. Previous studies assessing patient outcomes following post-discharge medication review services have found conflicting results. The HOMER trial questioned the value of the service as the intervention group had an increased hospitalization rate at six months post-discharge [18]. Similarly, Barker *et al. *trialed a home-based, post-discharge medication review service in patients with congestive heart failure (CHF) and found that patients in the intervention group had significantly longer CHF hospital stays, incidence rate ratio = 2.34 (*P *< 0.001) [19]. There were no statistical differences in the other two primary outcomes of death and hospital readmission rates. Conversely, Stewart *et al. *were able to demonstrate both a short- and long-term benefit by reducing hospital readmissions and mortality, following a post-discharge service targeted at a population that was suspected to be at a high risk for readmission [20,21]. While the goal of reducing healthcare costs through fewer and shorter hospitalizations appears appropriate, this outcome may not be a suitable measure for a service focusing specifically on medication management. A review by Benbassat *et al. *questioned the validity of hospital readmissions as a marker for quality care and highlighted that the length of hospital stays, readmission rates, and death appear to be mostly predicted by unmodifiable causes, such as age, disease severity, and co-morbidity [22]. The authors concluded by highlighting the importance of improving other 7clinical outcome measures, such as adherence to guideline-based therapy and improving patient's self-management abilities.

Although the interventions trialed in their studies included a significant focus on improving medication adherence, neither Holland *et al. *nor Barker *et al. *measured changes in this outcome. As such, both authors were left to speculate over this outcome and how it may have affected hospital readmission rates. Conversely, Stafford *et al. *conducted a prospective, non-randomized, controlled cohort study of a pharmacist-led service aimed at improving warfarin therapy post-discharge and, while there were no significant changes in readmission or death rates over the 90-day follow-up period, the intervention was associated with a reduced rate of adverse bleeding events from warfarin therapy, 5.3% *vs*. 14.7% (*P *= 0.03) and increased persistence with therapy, 95.4% *vs*. 83.6% (*P *= 0.004) [23]. The pharmacists involved in this trial were HMR-accredited, but also received additional education, specific to the needs of patients taking warfarin [24]. This ability to focus the HMR service on specific patient groups has been proposed as an avenue for improvement of the HMR service and this warrants further attention through future research [15]. We have considered both of these aspects in the modification of the service planned for this trial and have outlined that the period following hospital discharge is an appropriate time to target ACS patients due to their risk for harm resulting from premature medication discontinuation [2,3]. Furthermore, we have developed a detailed education program to help pharmacists address ACS-specific issues and encourage patient behavior change by using a motivational approach to the HMR patient interview.

### Objectives

In this study, we aim to investigate the effect of an adaptation of the currently available HMR service on guideline-based medication adherence and persistence following ACS. The service will be directed towards the needs of ACS patients by educating the accredited pharmacists involved about ACS-specific patient issues and through improving the continuum of care by providing consistent information in a structured HMR referral letter. As such, the trialed service will be termed a directed Home Medicines Review (dHMR). This is the first trial in a series of studies entitled 'Medication Reviews ReDirected (MedReDi)' which refers to the plan for future research in this area, following a similar theme of minor adjustments to the currently existing HMR service, so that patients with other specific illnesses can be targeted through new dHMR services. The overall intention will be to help patients become more familiar and competent with their medication management aspect of therapy - more simply, helping patients become 'MedReDi'.

For the purpose of this study, guideline-based ACS medication therapy refers to the four medication classes as supported by ACS guidelines [25-27]. These medication classes include: angiotensin-converting enzyme (ACE) inhibitors/angiotensin II receptor blockers (ARBs); antithrombotics (aspirin and/or clopidogrel/prasugrel and/or warfarin); beta-blockers; and statins.

## Methods/Design

### Overview

**Figure 1:**
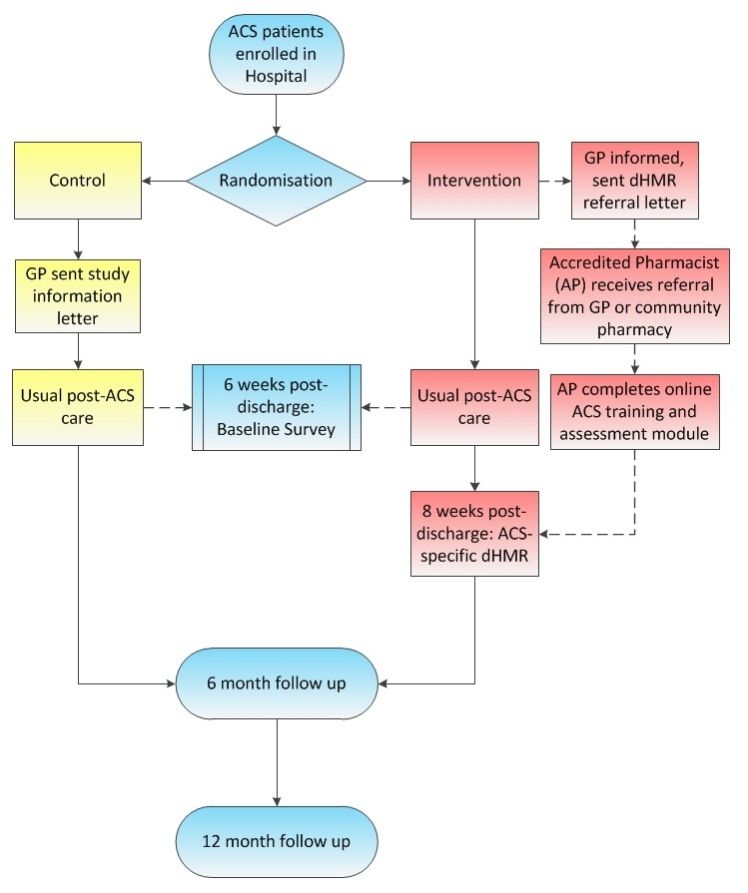
**Trial protocol overview**. ACS: Acute Coronary Syndrome; dHMR: Directed Home Medicines Review; GP: General Practitioner; AP: Accredited Pharmacist.

**Figure 2:**
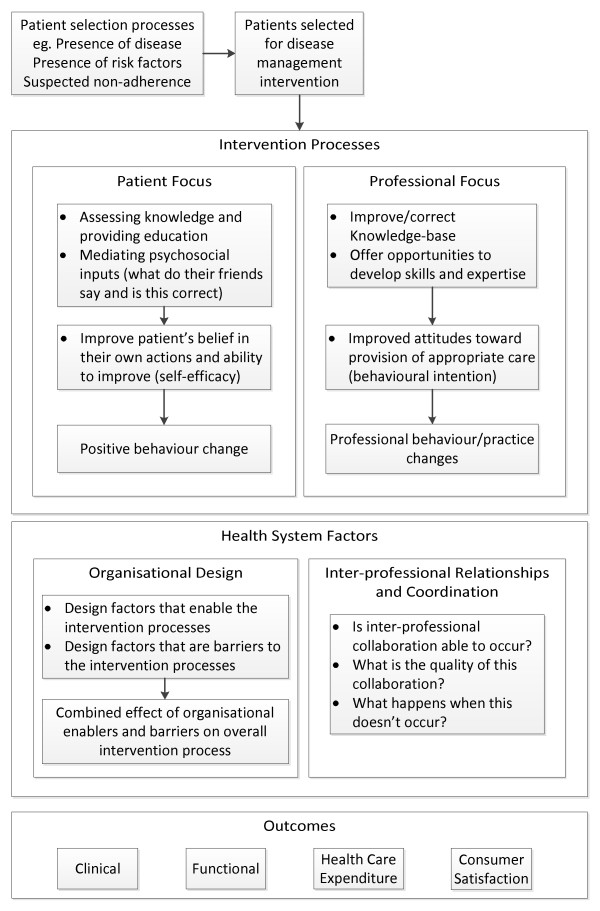
**Summary of the Lemmens *et al. *framework **[28]. This representation of the 'Evaluation model for disease-management programs' has been adjusted slightly from the originally produced model to better reflect the points of the framework that we consider relevant to the HMR service (shown in further detail through Figure 3). It is important to recognize that this framework highlights the importance of considering patient-related factors, professional-related factors, and health-system factors in both the design and evaluation of interventions targeted toward improving the management of chronic diseases. These considerations have been particularly important throughout the development of this trial.

**Figure 3:**
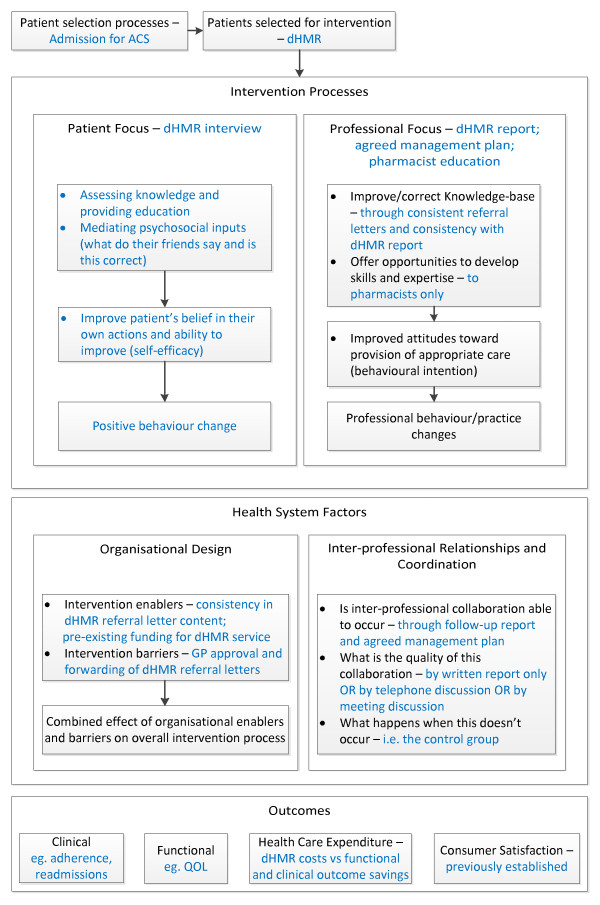
**How the proposed dHMR service addresses the specific components of the Lemmens *et al. *framework **[28]. ACS: Acute Coronary Syndrome; dHMR: Directed Home Medicines Review; GP: General Practitioner; QOL: Quality Of Life. The blue text highlights the areas of the framework where we are investigating the effect of a dHMR with specific detail provided for clarification.

We will conduct a randomized controlled trial comparing the dHMR service delivered at two months post-discharge to usual care following an ACS admission. Changes in the pharmacist referral process and an ACS-specific education package to be completed by the study pharmacists make this program different to the standard HMR service. The primary outcome will be the proportion of patients who are adherent to a complete, guideline-based ACS medication regimen at six and twelve months post-discharge. Patients will be enrolled in hospital during an admission for ACS. Figure 1 provides an overview of the trial protocol. The design of the intervention and monitoring system has been based on a conceptual framework for the standardized evaluation of chronic disease management interventions, as developed by Lemmens *et al. *[28]. Figure 2 is a summary of this framework and Figure 3 is a comparison of how the components of this trial compare against the Lemmens *et al. *framework.

This representation of the 'Evaluation model for disease-management programs' has been adjusted slightly from the originally produced model to better reflect the points of the framework that we consider relevant to the HMR service (shown in further detail in Figure 3). It is important to recognize that this framework highlights the importance of considering patient-related factors, professional-related factors, and health-system factors in both the design and evaluation of interventions targeted toward improving the management of chronic diseases. These considerations have been particularly important throughout the development of this trial

### Setting

Patient enrolment and dHMR referral will be from the two major tertiary referral hospitals in Tasmania, Australia. The Royal Hobart Hospital (RHH) is a 490-bed hospital with an 8-bed coronary care unit (CCU), percutaneous coronary intervention (PCI), and coronary artery bypass grafting (CABG) facilities, and accepts patients from across the state for CABG referrals [29]. The Launceston General Hospital (LGH) is a 300-bed hospital with a 4-bed coronary care unit and PCI facilities [30]. Both hospitals run cardiac rehabilitation programs that are offered to all ACS patients. Patients admitted to these centers generally receive verbal and written education regarding lifestyle changes following ACS from nurses, and medication counseling from nurses or pharmacists, prior to discharge.

### Inclusion/Exclusion criteria

All patients aged 18 to 80 years with a primary diagnosis of ACS presenting at the RHH and LGH will be considered for enrolment into the trial. The wide age range has been chosen to detect differences in the discharge management of patients across different age groups and to examine the suitability of the dHMR intervention across different age groups. The former problem has been previously recognized as an area for further interventional focus by Alexander *et al. *in the CRUSADE trial [31] and the latter has been recognized as an area requiring further investigation through a qualitative review of the HMR service [15].

Patients will be excluded from trial enrolment if they: are not returning to their home following hospital discharge (as this is a requirement of the existing HMR service); are non-Medicare eligible (for example, not a permanent Australian resident); have been diagnosed with a level of cognitive impairment such that the process of informed consent may be obscured; or have been diagnosed with a malignancy that is expected to be terminal within 12 months.

### Randomization

We will use a computer-generated random sequence to provide the randomization coding. Patients will be randomized to the control or intervention groups following computerized recognition that the patient meets the inclusion criteria and has consented to trial involvement. The correct entry of this information into the central trial database will then enable the enrolling researcher to unlock the randomization status for that participant.

### Controls

Following randomization to the control group, patients will be offered the usual care processes involved with post-discharge management of ACS in Australia. Through the public health system, patients are able to attend a cardiac rehabilitation program at their local hospital as well as a cardiologist follow-up appointment, usually occurring at one month following discharge. The cardiac rehabilitation programs from the two different hospitals may vary slightly in their level of physical activity, but the material covered in their information sessions is similar.

### Intervention

Patients in the intervention group will receive a dHMR at approximately two months following discharge as well as usual care. The time of two months post-discharge was selected as previous studies of ACS patients have demonstrated that the period between one and three months post discharge is when patients are most vulnerable for medication discontinuation [2,3,32]. This suggests that a service addressing both the educational and clinical management aspects of therapy may be most beneficial when offered in this time period.

Based on the existing funding arrangements and recommended reasons for referral, the HMR service is currently available in Australia for all ACS patients following hospital discharge [16]. The uptake of this service, however, may be largely limited by the absence of HMR referral systems in this transitional period. Furthermore, the currently available service could be tailored to maximize the potential for benefit from this service in the ACS population. We propose to add to the existing HMR service in several ways, making the proposed dHMR service more directed toward the ACS population.

Typically, HMRs are ordered by a referral letter generated by the patient's GP. Referral letters may vary significantly in their level of detail, potentially leaving the pharmacist with little direction prior to the patient interview. This concern was highlighted as a potential downfall of the service in a qualitative review that was conducted in 2008 [15]. To address this issue, the enrolling researchers will use a specifically designed database that allows baseline data collection to automatically populate sections of the HMR referral. The referral letter will still be sent to the GP for approval and addition of any further information, such as medications that have changed since discharge. GP approval is a requirement for payment under the existing public service arrangements, and having the GP engaged with the intervention early should increase their willingness to participate. This new approach, however, relieves the GP from having to complete most of the referral data and allows for simple addition of only useful information. The GP may also find the information on this referral letter useful when they receive the letter, as previous research has demonstrated that standard discharge letters following ACS admissions do not always contain a sufficient level of information and sometimes take too long to reach the GP [3].

In addition to the specific referral letter, the service will be further directed by offering accredited pharmacists across Tasmania an online education and assessment package. In order for a pharmacist to complete an interventional dHMR according to the trial protocol, they will be required to have a current accreditation status and have successfully completed the education and assessment package. Upon completion of the education and assessment package, accredited pharmacists will gain recognition of continuing professional development and an AUD$50 reimbursement for their time.

### Developing the education and assessment package

Within our trial, accredited pharmacists are appropriately poised to deliver education to patients at the HMR interview and to GPs through the HMR reports. Addressing both the patient and their GP in this fashion has been recognized as an important factor to increase the chance of improving patient outcomes through chronic disease management interventions [28,33,34]. Further education and specialization of accredited pharmacists in the area of ACS secondary prevention should increase the chance of the current service utilizing these opportunities for follow-on education.

The accredited pharmacist education and assessment package will consist of five online lectures, as well as several separate resource documents for pharmacists to refer to at any point throughout the HMR service. The five online lecture topics are: an introduction to the trial and the evidence to practice gaps for ACS; background information about the hospital management of ACS; the importance of cardiac rehabilitation and lifestyle modifications; evidence-based ACS medication management; and methods for improving adherence to therapy following ACS. The separate resource documents include a 'chest pain action plan', information on the use of short-acting nitrates, and links and referral to specific material available from the National Heart Foundation, Australia. The material will be assessed using case-based examples with multiple-choice questions (pass mark of 75%). The full details of the development of the education and assessment package will be published at a later stage.

### Outcomes

All outcomes will be measured using blinded assessment processes at the two study endpoints of six and twelve months post-discharge. Each outcome will be a comparison between the control and intervention groups. A proposed conceptual framework for the standardized evaluation of chronic disease management interventions has been considered in the selection of the trial outcomes and further trial evaluation [28]. Using a framework to guide outcome selection has not only helped to ensure that we are measuring the important clinical outcomes from the intervention, but also highlighted the individual steps of the intervention that require monitoring. Monitoring these individual steps, such as pharmacist detection of drug-related problems and GP acceptance of pharmacist recommendations, is important to measure the level of alignment between the theoretical plan and the practical application of the intervention. Such detailed monitoring and outcome reporting may also elucidate areas of practice worthy of further focus in future research.

### Primary outcome

The primary outcome will be the proportion of patients who are adherent to a complete, guideline-based ACS medication regimen at the trial endpoints. The accepted definition of adherence for this trial will be a medication possession ratio (MPR) of 80% to 120% for all guideline-based medications, which is a commonly accepted cut-off for this measure [35-39]. MPR will be determined by dividing the total number of days of medication supplied by the total specified time period. This value can be multiplied by 100 in order to be reported as a percentage of the time that the patient had medication available to take, a commonly used surrogate measure of adherence. During the baseline interview, we intend to record all possible pharmacies that a patient may attend in order to make the dispensing records complete. If, however, there is a significant shortfall in the completeness of dispensing records, we will use self-report of medication adherence as an alternate measure. The four-item Morisky adherence questionnaire will be used for this purpose [40]. This is a well-validated instrument, being used previously with good predictive validity in patients at risk of cardiovascular disease [40,41]. In the case where a patient has a clearly documented contraindication to one of the four guideline-based medication classes, their medication regimen may still be considered 'complete' provided they are taking all other guideline-based medications. For example, a patient with asthma not taking a beta-blocker but still taking aspirin, an ACE inhibitor, and a statin, will be considered in the same group as those prescribed all four medications. This primary outcome comprehensively assesses the overall impact of the intervention on both GP prescribing behaviors and patient medication-taking behaviors. In order to separately measure the recognized problem of persistence with guideline-based medications, we will complete a secondary analysis of the primary outcome in the group of patients who are prescribed a complete, guideline-based medication regimen at the time of discharge from hospital.

### Secondary outcomes

Secondary outcomes will include hospital readmission rates, length of hospital stays, changes in quality of life, cardiac rehabilitation completion rates, smoking cessations rates, and mortality. Hospital readmissions and mortality will be further categorized as ACS-related or due to other causes. Quality of life will be measured at six weeks post-discharge as a baseline, pre-dHMR measure and again at both study endpoints. The Euroqol 'EQ-5D 3 L' [42] will be used to measure general quality of life and the Seattle Angina Questionnaire (SAQ) [43] will be used to measure cardiac-specific quality of life. Both instruments have been validated when administered individually [43-46] and in combination [47] to assess quality of life in patients with coronary heart disease. In addition to a self-report of smoking status at baseline and the study endpoints, we will be further categorizing each smoker's dependence on cigarette smoking using the Fagerstrom Test for Nicotine Dependence (FTND) [48].

### Process outcomes

**Figure 4:**
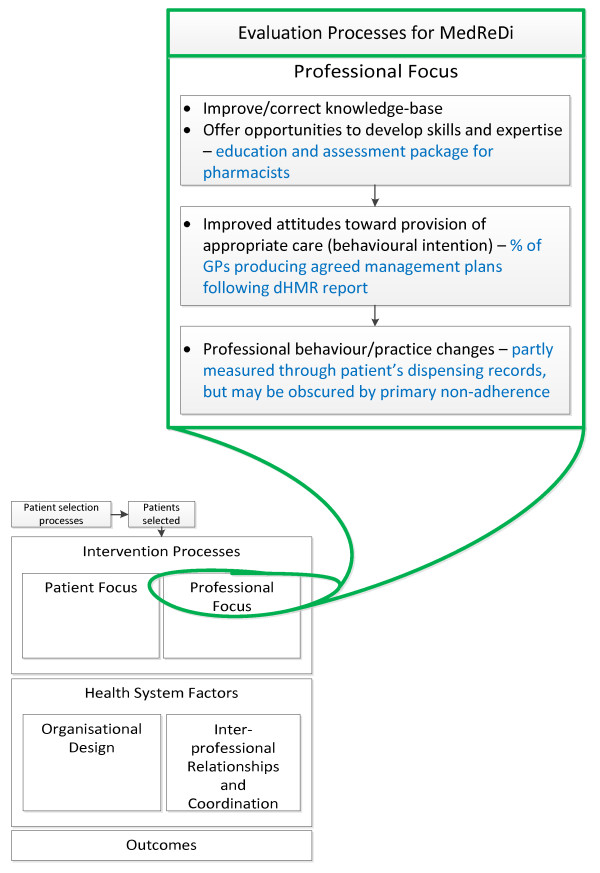
**Evaluation of the professional-focused components of the intervention **[28]. The blue text in this figure highlights where the professional focused components of the intervention fit into the Lemmens *et al. *framework. dHMR: Directed Home Medicines Review.

**Figure 5:**
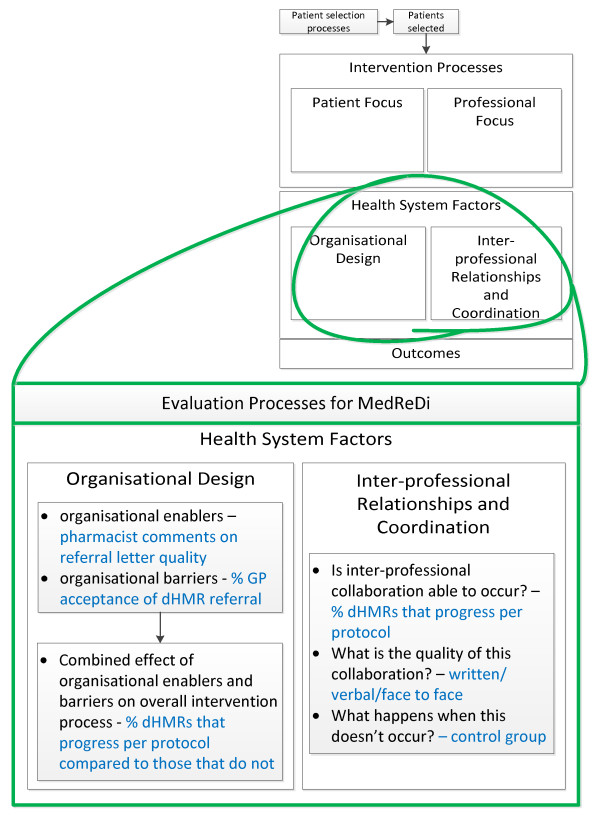
**Evaluation of the health system's impact on the implementation of the intervention **[28]. The blue text in this figure highlights how the local health-system structure may affect the implementation of the intervention described within this trial protocol. Again, the relevant points raised by the Lemmens *et al. *framework have been considered. dHMR: Directed Home Medicines Review.

In addition to reporting the important clinical outcomes that may be affected by the intervention, it is also recognized that there are many individual components of such interventions that can be measured and may highlight barriers or enablers to the overall success of the intervention [28]. From the professional side of the dHMR, it will be important to measure GP acceptance of the intervention through approval of dHMR referrals, pharmacists' recognition of drug-related problems, the clinical relevance of the drug-related problems identified, and GP acceptance of pharmacists' recommendations. Figures 4 and 5 represent our plans to measure the effect of these professional and organizational barriers on trial implementation.

**Figure 6:**
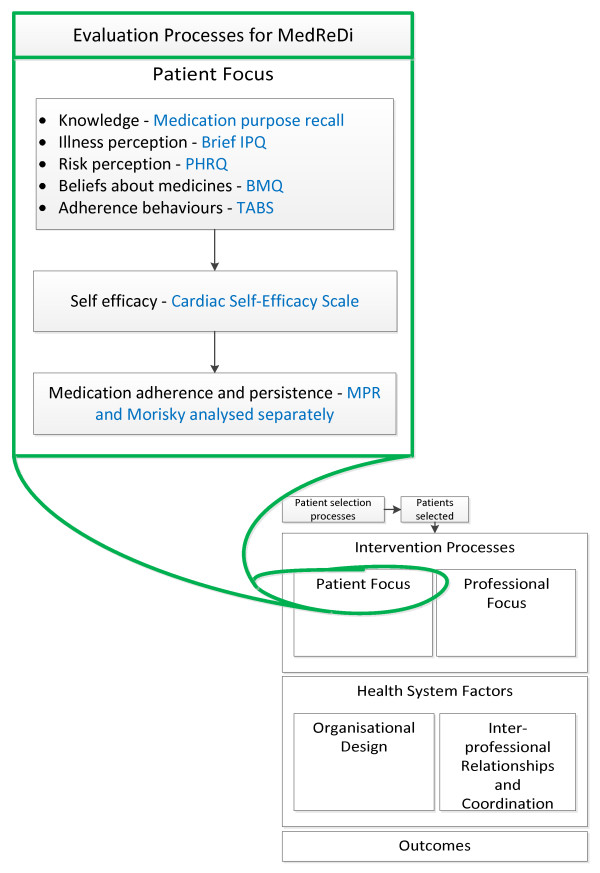
**Evaluation of the patient focused component of the intervention **[28,40,49-54]. This figure highlights the comprehensive evaluation that we have designed for the patient-focused component of the intervention. IPQ: Illness Perception Questionnaire; PHRQ: Perceived Health Risk Questionnaire; BMQ: Beliefs About Medicines Questionnaire; TABS: Tool for Adherence Behavior Screening; MPR: Medication Possession Ratio.

From the patient's perspective it is important to consider, firstly, how the dHMR process may influence psychological variables that have been previously recognized as relevant to adherence behavior, as well as how strongly these variables correlate to the outcome of adherence within this particular trial setting. The concepts we have chosen to consider and the validated instruments that will be used to evaluate each construct are detailed in Figure 6.

The concepts of knowledge [55-57], illness perception [49,58], beliefs about medications [59,60], and self-efficacy [50,61] have all been studied separately in coronary heart disease populations. In developing the conceptual framework for the evaluation of chronic disease interventions, however, Lemmens *et al. *recognized the important interrelations between these concepts and how this can affect the ultimate outcome of patient behavior change [28]. Risk perception [51] and adherence-specific behaviors [52] are two lesser studied concepts that may also affect or predict adherence and, as such, have been added to our model of assessment. As discussed, we consider it important to measure and report on the effect of the dHMR on these individual concepts, as well as the validity of the individual concepts as predictors for adherence within this trial setting. Again, the latter has been investigated on an individual basis for some concepts, but the different trial setting and the inclusion of further variables may result in alternate outcomes. The full details of the predictors of adherence recognized in our ACS population will be published elsewhere. The following is a brief summary of the questionnaires selected for this purpose: to assess medication knowledge we have selected recall of individual medication purpose as adapted from Hope *et al. *[53]; for illness perception we have selected the eight quantitative items of the Brief Illness Perception Questionnaire (Brief IPQ) [49]; for risk perception we have selected the Perceived Heart Risk Questionnaire (PHRQ) [51]; for beliefs about medications we have selected eight items from the Beliefs About Medicines Questionnaire (BMQ) [54] and developed two items to assess the impact of cost 'The cost of my medications makes it difficult for me to take them regularly' and 'Medications are not good value for money' which will be assessed on the same 5-point Likert scale as the BMQ questions; for self-efficacy we have selected seven items from the Cardiac Self-Efficacy Scale [50]; for adherence-specific behaviors we have selected the Tool for Adherence Behavior Screening (TABS) [52].

Further detail on the reasoning behind each questionnaire's selection and the reasons for abbreviation of full questionnaires, where relevant, will be discussed in a future publication on the predictors of adherence. All of these questionnaires have been validated in populations of patients with coronary heart disease.

In addition to these questionnaires it is important to note that for the purpose of testing for predictors of adherence, we will be considering two separate models. In the first model, adherence will be measured by the MPR and in the second model, adherence will be measured by the Morisky Adherence Questionnaire [40]. Both measures have individual benefits and limitations to their use, but have been extensively validated in cardiac settings [35,40,41,62,63]. It was considered important to include the second model using a questionnaire to assess adherence in order to maintain consistency with the reliance on questionnaires as the method of assessment for all concepts. The complete questionnaire set chosen for this study can be viewed in Additional file 1.

### Sample size

We are aiming to detect a change in the primary outcome of 15% between the control and intervention groups. This predicted change is based on the results of the DMACS study, whereby a 12% post-intervention improvement was observed on the proportion of patients taking the same four guideline-based medications at discharge [3]. To detect a change of 15%, assuming a control group result of 45%, a power to detect a difference of 80% with alpha = 0.05, we require a minimum sample size of 186 patients per group. To account for an approximate dropout rate of 20%, we aim to enroll 465 patients in total.

### Data collection

Baseline data collection will occur in hospital at the time of enrolment, immediately post-discharge from hospital and at six weeks post-discharge - when the participants will be sent their first questionnaire set. The in-hospital baseline data collection will include the recording of traditional coronary risk factors, as well as the 'cholesterol' version of the INTERHEART Modifiable Risk Score (IHMRS) [64]. This will allow for baseline comparison of risk between the two groups. Although not validated to calculate risk in a population of patients following a coronary event, the IHMRS requires the recording of risk factors in a way which emphasizes their correlation to their level of actual risk and this remains relevant, regardless of the patient's degree of heart disease. For example, the IHMRS requires the recording of smoking status into several cutoffs depending on the number of cigarettes smoked per day, which correlates independently to heart attack risk, as opposed to simply recording cigarette smoking status in an arbitrary fashion, which is of lesser value with regard to risk comparisons. The final baseline data collection point of six weeks post-discharge for questionnaire mail-out reflects our intention to gauge each patient's status on each of the questionnaires in the time directly before the HMR and not while in hospital, as factors, such as adherence behavior, may be very high in hospital but may significantly decline over time following discharge [2,3,14,65,66]. Patients in the intervention group will be asked to ensure that they complete their questionnaire before their pharmacist visit and pharmacists will record whether or not this has happened as reported by the patient. Patients in the control group will be given a reminder telephone call if their questionnaires have not been returned by three weeks from the mail-out date.

Accredited pharmacists' successful completion of the education and assessment package will be required prior to starting any trial dHMR. This will be recorded through an online system, specifically designed to guide each pharmacist through to completion of this package, while also monitoring their usage levels of the education website via separate log in codes. Although completion of the package is encouraged by an AUD$50 honorarium, program completion cannot be enforced beyond this level and those patients receiving a dHMR by a pharmacist who has not successfully finished the education and assessment package prior to the dHMR interview will be excluded from the secondary on-treatment analysis, as discussed in the 'statistical analysis' section.

Following the dHMR interview, data collection starts with the pharmacist's dHMR report and the agreed, patient-GP management plan. Collection of these documents will allow for assessment of pharmacists' recognition of drug-related problems, the recommendations made to improve these problems, and the GPs' acceptance of these recommendations, as described in Figures 4 and 5. Telephone follow-up will be considered two months after the interview if these reports and management plans have not been received. Recent amendments to the Medicare-funded HMR reimbursement process have strongly increased the requirement that an agreed management plan be formulated following all HMRs, which should help facilitate this data collection point.

Six and twelve months post-discharge follow-up will include requests to community pharmacies for patient dispensing records, hospital register checks for readmissions and length of stays, a death's registry check for mortality, and questionnaire mail-out with a three-week phone call reminder.

### Statistical analysis

All baseline variables will be compared between the control and intervention groups using independent samples t-tests for continuous variables and chi-squared analysis for categorical variables. All outcomes will be reported as descriptive comparisons between the control and intervention groups, with significance reported at alpha = 0.05. Where primary outcomes reach statistical significance, any noted difference in the baseline group comparison will be tested by univariate comparison with stepwise addition into a multivariate model for those with univariate significance of *P *< 0.1. Changes in each group's questionnaire results between baseline and study endpoints, as well as differences between intervention and control group's questionnaire results, will be reported. Further correlational investigations between questionnaire results and adherence will be reported elsewhere. As there are several potential barriers that may prevent a patient randomized to the intervention group from receiving a dHMR according to the protocol, we will use both an intention-to-treat analysis as well as an on-treatment sensitivity analysis.

### Ethical approval

This trial has received ethical approval through the Tasmanian Health and Medical Human Research and Ethics Committee. Approval number: H11821. All participating patients will provide informed consent.

## Discussion

Recent evidence supports the current need for an intervention directed at improving the prescription of, and patients' adherence to, guideline-based therapy in the period following ACS hospital discharge [2,3]. This trial aims to achieve this outcome using an intervention that is based on only minor adjustments to a currently existing and funded service, such that any success derived from this trial may be easily translated into regular practice. The potential for providing directly applicable benefits to society by using this proposed method was recognized in a recent editorial by Davidson and Macdonald [67]. Should our intervention lead to an improvement in the primary outcome, the inherent sustainability of the intervention in a real world environment is one of its major strengths.

As mentioned through this paper, the DMACS study demonstrated significant improvements in prescribing at discharge, but these improvements were almost completely lost at three months post-discharge [3]. Therefore it could be proposed that, ideally, the intervention described within this trial would occur in conjunction with a similar quality improvement study. This would ensure that both the in-hospital and post-discharge aspects of management were addressed together, allowing for an even greater opportunity to demonstrate sustained improvements. As the specific benefits from a dHMR service directed at ACS patients are unknown, however, it is logical to trial this service first, before considering its position in conjunction with other interventions. Australia's National Prescribing Service offers kits to allow ongoing drug use evaluations similar to that of the DMACS study, therefore a trial combining an in-hospital intervention and a post-discharge dHMR may be feasible for future research, should the current trial lead to success worthy of this follow-up.

Although based on an existing, Medicare-funded service, the treatment protocol faces several potential barriers to success. Largely, this includes interprofessional barriers, such as GP approval of the dHMR referrals and GP acceptance of pharmacists' recommendations. The former point will be aided by the enrolling researcher contacting the GP once randomization has been revealed. Although GP approval of dHMR referrals may affect the uptake of the intervention in this trial, the use of a consistent and informative referral letter and a telephone conversation to support this letter, could prove to be advantageous over the current systems for initiating standard HMR referrals, which may not always be this well-structured. The other barrier of GPs not accepting pharmacists' recommendations has been raised due to previous research showing that GPs only action approximately 45% of pharmacist's recommendations following HMRs [68,69]. The educational material provided to accredited pharmacists completing this trial, however, should help guide pharmacists to make recommendations which will be relevant post-ACS and simple to implement. Furthermore, as the education package has been based on the latest evidence in ACS management, even if GPs do not accept all of the pharmacists' recommendations, they should still be gaining useful information from the dHMR reports. This may lead to improved ACS management, irrespective of the rate at which GPs accept pharmacists' individual recommendations.

Having the pharmacists complete the online training package may also be another barrier to the successful implementation of the trial protocol. The education and assessment package should take approximately three hours to complete and will be rewarded with recognition of continuing professional development and an AUD$50 honorarium. We hope that these incentives and the easy-to-use format of the material will help facilitate the completion of the package. Through careful consideration of the potential barriers recognized above, the dHMR processes that we have developed have transformed these barriers into opportunities to improve the implementation of the intervention.

We have designed a trial to test the effect of minor changes to an existing, funded model of intervention to target the needs of a high-risk population. The conceptually derived intervention, based on the Lemmens *et al. *framework, accommodates funding and other health-system-related barriers which are critical for successful implementation. The intervention will be assessed by observing changes in medication adherence which is an outcome that appropriately matches the aim of the intervention. We have outlined the potential benefit from the application of this intervention on a worldwide scale, should the intervention demonstrate positive outcomes in the trial population of Australian ACS patients.

## Trial status

The trial has not yet started recruiting. The expected start time is early 2012.

## Abbreviations

ACE inhibitor: Angiotensin-Converting Enzyme Inhibitors; ACS: Acute Coronary Syndromes; AP: Accredited Pharmacist; ARBs: Angiotensin II Receptor Blockers; BMQ: Beliefs About Medicines Questionnaire; Brief IPQ: Brief Illness Perception Questionnaire; CABG: Coronary Artery Bypass Graft; CCU: Coronary Care Unit; CR: Cardiac Rehabilitation; dHMR: Directed Home Medicines Review; DMACS: Discharge Management of Acute Coronary Syndrome; FTND: Fagerstrom Test for Nicotine Dependence; GP: General Practitioner; HMR: Home Medicines Review; IHMRS: INTERHEART Modifiable Risk Score; LGH: Launceston General Hospital; MPR: Medication Possession Ratio; PCI: Percutaneous Coronary Intervention; PHRQ: Perceived Health Risk Questionnaire; QOL: Quality Of Life; RHH: Royal Hobart Hospital; SAQ: Seattle Angina Questionnaire; TABS: Tool for Adherence Behavior Screening

## Competing interests

All of the authors declare no conflict of interest in the outcomes or conduct of this research.

## Authors' contributions

DDLB, LS, LREB, and GMP have contributed equally to the study design process. PMD has contributed to the study design, with particular input on study evaluation and questionnaire selection. RLC has contributed to the study design. DDLB drafted this manuscript. All authors have contributed to the revision of the manuscript and approved of the final copy.

## Authors' information

Mr Daniel DL Bernal is a current PhD candidate at the School of Pharmacy, UTAS, Tasmania, Australia. Ms Leanne Stafford has submitted her PhD on the topic of post-discharge medication review services to improve the use of warfarin therapy and currently works as a lecturer at the School of Pharmacy, UTAS, Tasmania, Australia. Dr Luke RE Bereznicki is the Deputy Head of School and Senior lecturer at the School of Pharmacy, UTAS, Tasmania, Australia. Dr Ronald L Castelino is a research fellow and lecturer at the School of Pharmacy, UTAS, Tasmania, Australia. Prof Patricia M Davidson is the Director of the Centre for Cardiovascular and Chronic Care at the UTS, Sydney, Australia. Prof Gregory M Peterson is the Head of School of Pharmacy and Director for UMORE, UTAS, Tasmania, Australia.

## Supplementary Material

Additional file 1 **MedReDi Patient Questionnaire Set **[40,49,55-59]. Click here for file
