# Supplementary material for: Home medicines reviews following acute coronary syndrome: study protocol for a randomized controlled trial
Source: Trials. 2012 Apr 2;13:30. doi: 10.1186/1745-6215-13-30 (PMC3349589; doi:10.1186/1745-6215-13-30)
Supplement: Additional file 1 — MedReDi Patient Questionnaire Set [40,49,55-59]. [file 1745-6215-13-30-S1.DOC]

**Home Medicines Review Following Heart Events**

**Participant Questionnaire Set**

**Questionnaire 1: Medication List and Medication Adherence**

| Patient Study Number: |  | Completed by? Patient / Carer |
| --- | --- | --- |

*Please collect together all your current medicines and list them below. Take a moment to notice the layout of the table – there is a separate section for the name, strength, dosing time, and number of tablets that you take. The final column is a short test of your medicine knowledge. You should try to answer this yourself, without any aids. Do your best, there is no penalty for any incorrect information. There is a second page if required.*

| Current Medication (brand or drug name) | Strength | Time when you take it? | How many you take? | What is it for? |
| --- | --- | --- | --- | --- |
| **Example:**  Prednisolone | 5mg | Morning | ½ | Arthritis |
|  |  |  |  |  |
|  |  |  |  |  |
|  |  |  |  |  |
|  |  |  |  |  |
|  |  |  |  |  |
|  |  |  |  |  |
|  |  |  |  |  |
|  |  |  |  |  |
|  |  |  |  |  |
|  |  |  |  |  |
|  |  |  |  |  |
|  |  |  |  |  |
|  |  |  |  |  |
|  |  |  |  |  |
|  |  |  |  |  |
|  |  |  |  |  |
|  |  |  |  |  |
|  |  |  |  |  |
|  |  |  |  |  |
|  |  |  |  |  |
|  |  |  |  |  |

Please place a cross **(X)** in **ONE** box that best applies to you. There is no right or wrong answer, we are looking for an honest answer.

|  | Yes | No |
| --- | --- | --- |
| 1. Do you ever forget to take your medication? |  |  |
| 1. Are you careless at times about taking your medication? |  |  |
| 1. When you feel better, do you sometimes stop taking your medication? |  |  |
| 1. Sometimes, if you feel worse when you take your medication, do you stop taking it? |  |  |

**Questionnaire 2**

Many people find a way of using their medicines that suits them. Here are some ways in which people have said they use their medicines. For each statement, please place a cross **(X)** in **ONE** box that best applies to you.

|  | Never | Rarely | Sometimes | Often | Always |
| --- | --- | --- | --- | --- | --- |
| 1. I get confused about my medication |  |  |  |  |  |
| 1. I have strict routines for using my regular medication |  |  |  |  |  |
| 1. I keep my medications close to where I need to use them |  |  |  |  |  |
| 1. I ensure I have enough medication so that I don’t run out |  |  |  |  |  |
| 1. I strive to follow the instructions of my doctors |  |  |  |  |  |
| 1. I make changes in the recommended management to suit my lifestyle |  |  |  |  |  |
| 1. I vary my recommended management based on how I am feeling |  |  |  |  |  |
| 1. I put up with my medical problems before taking any actions |  |  |  |  |  |

**Questionnaire 3**

Sometimes people have different thoughts or feelings about how much the changes they make to their lifestyle or the medications that they take will improve their condition or illness. For each statement, please place a cross **(X)** in **ONE** box that best applies to you.

|  | Not at all confident | Somewhat Confident | moderately confident | Very Confident | Completely confident |
| --- | --- | --- | --- | --- | --- |
| 1. How confident are you that you can control your chest pain by changing your activity levels |  |  |  |  |  |
| 1. How confident are you that you can control your chest pain by taking your heart medication |  |  |  |  |  |
| 1. How confident are you that you know when you should call or visit your doctor about your heart disease |  |  |  |  |  |
| 1. How confident are you that you know how to take your heart medication correctly |  |  |  |  |  |
| 1. How confident are you that you can maintain your usual activities at home with your family |  |  |  |  |  |
| 1. How confident are you that you can maintain your usual activities at work |  |  |  |  |  |
| 1. How confident are you that you can get regular aerobic exercise (work up a sweat and increase your heart rate) |  |  |  |  |  |

**Questionnaire 4**

We recognise that not everyone feels the same way about medicines. This questionnaire will help us understand your thoughts about taking medicine. Please indicate your thoughts or beliefs about medicines by placing a cross **(X)** in **ONE** box per statement that best indicates the way you feel about taking medicines.

|  | Strongly disagree | Disagree | Neither agree nor disagree | Agree | Strongly agree |
| --- | --- | --- | --- | --- | --- |
| 1. Without my heart medication I would be very sick |  |  |  |  |  |
| 1. Having to take my heart medication worries me |  |  |  |  |  |
| 1. Doctors prescribe too many medications |  |  |  |  |  |
| 1. Most medications are addictive |  |  |  |  |  |
| 1. My heart health in the future will depend on my heart medication that I am taking currently |  |  |  |  |  |
| 1. I sometimes worry about the long-term side effects of my heart medication |  |  |  |  |  |
| 1. Natural remedies are safer than medications |  |  |  |  |  |
| 1. Medications do more harm than good |  |  |  |  |  |
| 1. The cost of my medications makes it difficult for me to take them regularly |  |  |  |  |  |
| 1. Medications are not good value for money |  |  |  |  |  |

**Questionnaire 5**

In order to gain an idea of how much you believe that your heart condition affects you, please circle **ONE** number per question that best indicates how you feel about your heart condition. This questionnaire extends over two pages.

| 1. How much does your heart condition affect your life? | | | | | | | | | | | | | | | | | | | | | | |
| --- | --- | --- | --- | --- | --- | --- | --- | --- | --- | --- | --- | --- | --- | --- | --- | --- | --- | --- | --- | --- | --- | --- |
| no affect at all | 0 | 1 | 2 | | 3 | | 4 | | 5 | | 6 | | 7 | | 8 | | 9 | | 10 | | severely affects my life | |
| 1. How long do you think your heart condition will continue? | | | | | | | | | | | | | | | | | | | | | | |
| a very short time | 0 | 1 | 2 | | 3 | | 4 | | 5 | | 6 | | 7 | | 8 | | 9 | | 10 | | forever | |
| 1. How much control do you feel you have over your heart condition? | | | | | | | | | | | | | | | | | | | | | | |
| absolutely no control | 0 | 1 | 2 | | 3 | | 4 | | 5 | | 6 | | 7 | | 8 | | 9 | | 10 | | almost total control | |
| 1. How much do you think your treatment can help your heart condition? | | | | | | | | | | | | | | | | | | | | | | |
| not at all | 0 | 1 | 2 | | 3 | | 4 | | 5 | | 6 | | 7 | | 8 | | 9 | | 10 | | extremely helpful | |
| 1. How much do you experience symptoms from your heart condition? | | | | | | | | | | | | | | | | | | | | | | |
| no symptoms | 0 | 1 | 2 | | 3 | | 4 | | 5 | | 6 | | 7 | | 8 | | 9 | | 10 | | many severe symptoms | |
| 1. How concerned are you about your heart condition? | | | | | | | | | | | | | | | | | | | | | | |
| not at all concerned | 0 | 1 | 2 | | 3 | | 4 | | 5 | | 6 | | 7 | | 8 | | 9 | | 10 | | extremely concerned | |
| 1. How well do you feel you understand your heart condition? | | | | | | | | | | | | | | | | | | | | | | |
| don’t understand at all | 0 | 1 | 2 | | 3 | | 4 | | 5 | | 6 | | 7 | | 8 | | 9 | | 10 | | understand very clearly | |
| 1. How much does your heart condition affect you emotionally? (For example, does it make you angry, scared, upset, or depressed?) | | | | | | | | | | | | | | | | | | | | | | |
| not at all | 0 | 1 | | 2 | | 3 | | 4 | | 5 | | 6 | | 7 | | 8 | | 9 | | 10 | | extremely affected |
| 1. How serious do you think your current heart condition is? | | | | | | | | | | | | | | | | | | | | | | |
| not at all serious | 0 | 1 | | 2 | | 3 | | 4 | | 5 | | 6 | | 7 | | 8 | | 9 | | 10 | | very serious |
| 1. How do you rate your chance of having the same, or developing the same heart problem again in your lifetime? | | | | | | | | | | | | | | | | | | | | | | |
| unlikely to happen again | 0 | 1 | | 2 | | 3 | | 4 | | 5 | | 6 | | 7 | | 8 | | 9 | | 10 | | almost certain to happen again |
| 1. Compared to other people of your same age and gender, how would you rate your chance of having the same heart condition again? | | | | | | | | | | | | | | | | | | | | | | |
| unlikely to happen again | 0 | 1 | | 2 | | 3 | | 4 | | 5 | | 6 | | 7 | | 8 | | 9 | | 10 | | almost certain to happen again |
| 1. How bad would it be for you if you were to have the same heart problem again? | | | | | | | | | | | | | | | | | | | | | | |
| not very bad at all | 0 | 1 | | 2 | | 3 | | 4 | | 5 | | 6 | | 7 | | 8 | | 9 | | 10 | | very bad |

Smoking Status

| Do you smoke cigarettes? | |
| --- | --- |
| 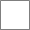Yes | 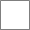No |

If you answered “Yes”, please complete the short questionnaire on the following page. If you answered “No”, you have completed this set of questionnaires. Thank you for completing this survey. Once you have completed the other survey marked on the front “EQ-5D”, please place the two questionnaires in the stamped, return envelope and place them in the mail.

**Questionnaire 6 (Cigarette Smokers Only)**

This questionnaire will tell us important information about your cigarette habits. Please place a cross **(X)** in **ONE** box per statement that best indicates your normal smoking habits.

| 1. How soon after you wake up do you smoke your first cigarette? | | | |
| --- | --- | --- | --- |
| 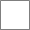Within 5 minutes | 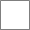6 – 30 minutes | 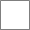31 – 60 minutes | 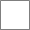After 60 minutes |
| 1. How many cigarettes a day do you smoke? | | | |
| 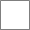10 or less | 11 – 20 | 21 – 30 | 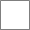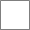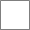31 or more |
| 1. Do you find it is difficult to refrain from smoking in places where it is forbidden (eg. In church, at the library, in cinemas, etc.)? | | | |
| 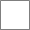Yes | | 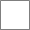No | |
| 1. Which cigarette would you hate most to give up? | | | |
| 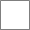The first one in the morning | | 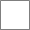All others | |
| 1. Do you smoke more frequently during the first hours after awakening than during the rest of the day? | | | |
| 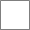Yes | | 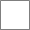No | |
| 1. Do you smoke if you are so ill that you are in bed most of the day? | | | |
| 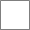Yes | | 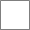No | |

Thank you for completing this survey. Once you have completed the other survey marked on the front “EQ-5D”, please place the two questionnaires in the stamped, return envelope and place them in the mail.
